# Tracing the sources of nutrients fueling dinoflagellate red tides occurring along the coast of Korea using radium isotopes

**DOI:** 10.1038/s41598-019-51623-w

**Published:** 2019-10-25

**Authors:** Hyeong Kyu Kwon, Guebuem Kim, Yongjin Han, Junhyeong Seo, Weol Ae Lim, Jong Woo Park, Tae Gyu Park, In-Seong Han

**Affiliations:** 1 School of Earth and Environmental Sciences/Research Institute of Oceanography, Seoul National University, Seoul, 08826 Republic of Korea; 2 Ocean Climate and Ecology Research Division, National Institute of Fisheries Science, Busan, 46083 Republic of Korea; 3 Southeast Sea Fisheries Research Institute, National Institute of Fisheries Science, Tongyeong, 53085 Republic of Korea

**Keywords:** Marine chemistry, Marine chemistry

## Abstract

It is a well held concept that the magnitude of red-tide occurrence is dependent on the amount of nutrient supply if the conditions are same for temperature, salinity, light, interspecific competition, etc. However, nutrient sources fueling dinoflagellate red-tides are difficult to identify since red tides usually occur under very low inorganic-nutrient conditions. In this study, we used short-lived Ra isotopes (^223^Ra and ^224^Ra) to trace the nutrient sources fueling initiation and spread of *Cochlodinium polykrikoides* blooms along the coast of Korea during the summers of 2014, 2016, and 2017. Horizontal and vertical distributions of nutrient concentrations correlated well with ^224^Ra activities in nutrient-source waters. The offshore red-tide areas showed high ^224^Ra activities and low-inorganic and high-organic nutrient concentrations, which are favorable for blooming *C. polykrikoides* in competition with diatoms. Based on Ra isotopes, the nutrients fueling red-tide initiation (southern coast of Korea) are found to be transported horizontally from inner-shore waters. However, the nutrients in the spread region (eastern coast of Korea), approximately 200 km from the initiation region, are supplied continuously from the subsurface layer by vertical mixing or upwelling. Our study highlights that short-lived Ra isotopes are excellent tracers of nutrients fueling harmful algal blooms in coastal waters.

## Introduction

The number of harmful algal blooms has been increasing gradually for decades around the world^1,2^. These blooms often involve serious economic impact of pollution and marine resources in the coastal areas, causing the massive mortality of aquaculture and wild fishes or shellfishes^3–5^. Amongst harmful algae, *Cochlodinium polykrikoides* is one of the most harmful dinoflagellates that causes critical damage to aquaculture in coastal waters, particularly in Asia, including Korea, Japan, Malaysia, and Philippines^4–7^. In Korea, *C. polykrikoides* is a dominant species causing harmful dinoflagellate blooms (hereafter red tides), and the area affected by red tides has expanded along the coastline^5^. Red tides usually initiate in the southern coast of Korea, and then spread to the western (Yellow Sea) coast by the Yellow Sea Warm Current or to the eastern (East Sea/Japan Sea) coast by the Tsushima Warm Current^5^. The magnitude (5800–48000 cells mL^−1^), duration (29–75 days), and initiation region (eastern or western coasts of Korea) of red tides have varied from 1995 to 2015. During this period, the fish kills caused by red tides have resulted in losses of US$ 1–67 million for Korea’s aquaculture industry^8^. However, there were no reports of the damage caused by red tides from 2016 to 2018 as the intensity of red tides diminished (<4500 cells mL^−1^) in early stages (<18 days), although the reason for this is unknown.

A number of studies have been conducted to determine the mechanisms for the outbreak and spread of red tides occurring along the southern coast of Korea. Yang *et al*.^9^ suggested that the outbreak of red tides in the southern coast of Korea is associated with the input of dissolved inorganic nitrogen (DIN) from the Chanjiang diluted water (CDW). On the other hand, Lee^10^ suggested that the massive outbreak of red tides in this region is led by the input of a large quantity of DIN following heavy rainfall events. More recent studies reported that the main source of nutrients in the southern coast of Korea is driven by submarine groundwater discharge^11–13^. Kim *et al*.^14^ and Lee *et al*.^13^ showed that the outbreaks of red tides in this region happen when dissolved inorganic nutrients are completely depleted and dissolved organic nutrients are abundant. However, the main source of nutrients fueling red tides in the study region (Tongyeong, the southeastern coast of Korea) is still poorly understood, and the supply of nutrients to the red-tide spread regions in the eastern coast of Korea is unknown.

**Figure 1:**
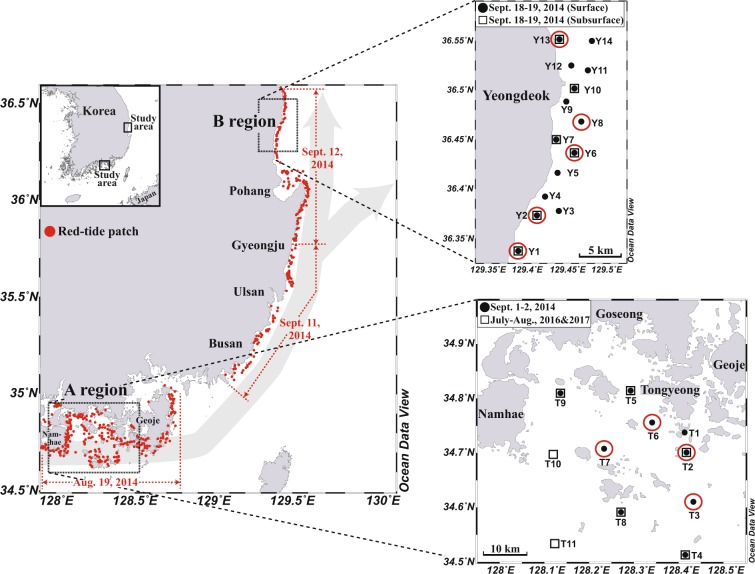
Maps showing sampling stations in the coasts of Tongyeong (A region) and Yeongdeok (B region), Korea. The gray filled arrow is a general concept of the spread of red tides based on a particle-tracking model and satellite remote sensing reported by Onitsuka *et al*.^6^, Choi *et al*.^32^, and Kim *et al*.^33^. The date indicates the timing of red-tide warning in 2014. The red open-circled stations denote the center of the red-tide patches. The station maps were created using Ocean Data View software version 5.1.7. (Schlitzer, R., Ocean Data View, odv.awi.de, 2018) and modified using Adobe Illustrator CS6 software version 16.0.0. (https://www.adobe.com).

Thus, in this study, we attempted to determine the characteristics and source of nutrients (1) in the Tongyeong coastal area in the southern sea of Korea where red tides are often initiated and (2) in the spread region (Yeongdeok) in the eastern coast of Korea, approximately 200 km from the initiation region. We measured short-lived Ra isotopes (^223^Ra and ^224^Ra), phytoplankton pigments, dissolved inorganic and organic nutrients in the red-tide regions of the southern coast and eastern coast of Korea during the summers of 2014, 2016, and 2017. In 2014, red tides initiated off Tongyeong on July 24 (~80 cells mL^−1^), and developed to a red-tide warning stage (>1000 cells mL^−1^) on August 19 (Fig. 1). The red tides expanded eastward, reaching as far as the coast of Yeongdeok in the eastern coast of Korea on September 12. The red tides lasted for the longest duration (79 days) since 1995 and resulted in fishery losses amounting to US$ 7 million^8^. However, there were no outbreaks of red tides in 2016 and 2017.

## Materials and Methods

### Study region

The study region is the coastal area of Tongyeong (A), the southern sea of Korea (Fig. 1). This region is one of the main regions where red tides initiate during summer. The water depth of this region is relatively shallow in the nearshore area (<10 m depth) and increases to ~50 m in offshore areas (30 km from the coastline). In this region, the open-ocean seawater belongs to the Tsushima Current, which is a tributary of the oligotrophic Kuroshio Current. During summer, this region is often affected by the low-salinity CDW^15^. Since there is no distinctive river in this region, groundwater discharge is known to be the major source of nutrients^11^. The water temperature and salinity in the surface layer of this region in August were average 24.8 °C and 32.23, respectively (http://www.meis.go.kr), which are favorable conditions for the growth of *C. polykrikoides*^16^.

### Sampling

Sampling was performed in A region in September 1–2, 2014 during the massive red-tide period (Fig. 1). Additional sampling was conducted in coastal waters off Yeongdeok (B) in the eastern coast of Korea, approximately 200 km from A region, which is downstream of A region, in September 18–19 to investigate the long-range spread of the red tides (Fig. 1). The seawater samples were taken from the visually-distinct centers of red-tide patches, as well as from visually non-affected stations for comparison (Fig. 1). In 2016 and 2017, the sampling was carried out with two week intervals in A region during July and August, but red tides did not occur (Fig. 1). Seawater samples for phytoplankton pigments, Ra isotopes (^223^Ra and ^224^Ra) and dissolved inorganic/organic nutrients were collected from the surface layer (0.5 m depth) of both regions. In addition, the subsurface water samples (10–20 m depth) were collected from B region, where the water depth approaches 90 m, with a submersible pump on shipboard. Salinity was measured *in-situ* with a portable multi-meter (Orion Star A329, Thermo Scientific, USA).

### Measurements of phytoplankton pigments, Ra isotopes, and nutrients

Seawater samples for pigments were filtered using glass-fiber filters (Whatman GF/F, 0.7 µm pore size) and stored at −80 °C until analysis. The pigments in the filter samples were extracted in 95% methanol with an internal standard canthaxanthin at 4 °C for 24 h in the dark. The extract was sonicated 5 min at low temperature (~5 °C) and then centrifuged at 1500 g for 10 min. The supernatants were filtered through a nylon syringe filter (0.2 µm pore size) in order to remove the remains of cell debris and filter. The extract was analyzed by using HPLC (Waters 2695) with a Waters Symmetry C_8_ column (4.6 × 150 mm, 3.5 μm particle size, 100 Å pore size). The procedures for HPLC analyses followed the method described in Zapata *et al*.^17^. The marker pigments were identified and quantified based on their retention time against authentic standards (DHI Inc. Denmark).

Seawater samples (~100 L) for Ra analysis were collected in polypropylene cubitainers and then passed through MnO_2_-impregnated acrylic fiber (MnO_2_-fiber) by gravity at a flow rate less than 1 L min^−1^ ^18^. In the laboratory, MnO_2_-fiber samples were washed using deionized water, and the water content was adjusted for measuring the activities of ^223^Ra and ^224^Ra on MnO_2_-fiber samples with a delayed coincidence counting system (RaDeCC)^18,19^. All the samples were measured within 4 days after the sampling. The purity of ^223^Ra and ^224^Ra was checked by subsequent measurements for their own decay rates. In general, the effect of ^228^Th was negligible.

Seawater samples for nutrient analysis were gently filtered through pre-combusted (450 °C, 4 h) GF/F (0.7 µm pore size) and stored at −20 °C until analysis. The concentrations of NH_4_^+^, NO_2_^−^, NO_3_^−^, and PO_4_^3−^ were measured using an auto nutrient analyzer (New QuAAtro39, Seal Analytical, Germany). Seawater reference material of nutrients (KANSO Technos, Japan) was used to verify the accuracy of nutrient concentrations. Here, dissolved inorganic nitrogen (DIN) was determined as the sum of NH_4_^+^, NO_2_^−^ and NO_3_^−^, and dissolved inorganic phosphorus (DIP) as PO_4_^3−^. Dissolved total nitrogen (DTN) and dissolved total phosphorus (DTP) were analyzed with an auto nutrient analyzer following persulfate oxidation (120 °C, 30 min)^20^. Deep-seawater reference materials of DTN (32–33 µM; University of Miami, USA) were run to verify the DTN analyses. The concentrations of dissolved organic nitrogen (DON) and dissolved organic phosphorus (DOP) were determined as the differences between DTN and DIN, and DTP and DIP, respectively.

## Results and Discussion

### Characteristics of phytoplankton pigments and nutrients in red-tide regions

**Figure 2:**
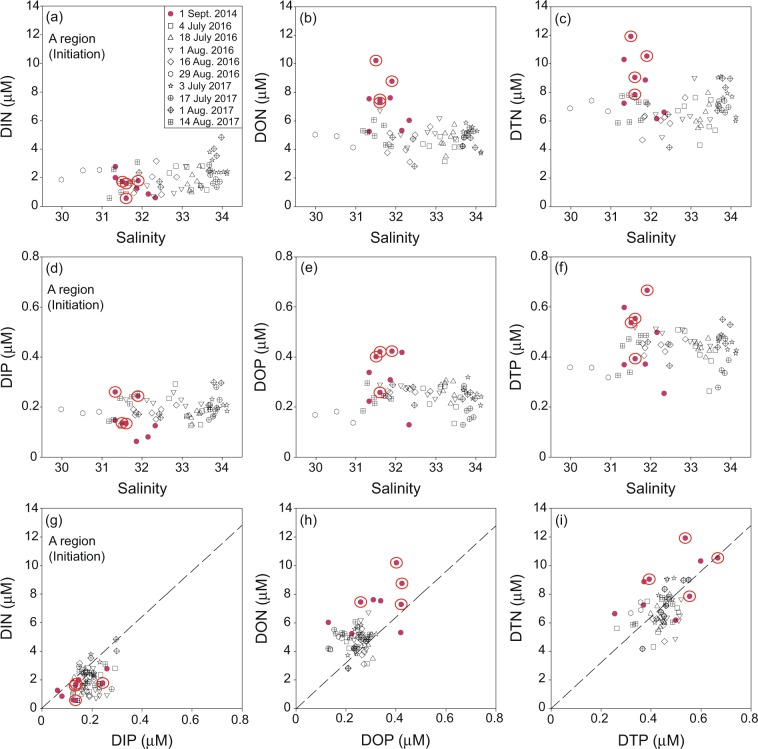
Scatter plots of salinities versus (**a**) DIN, (**b**) DON, (**c**) DTN, (**d**) DIP, (**e**) DOP, (**f**) DTP concentrations, (**g**) DIN versus DIP concentrations, (**h**) DON versus DOP concentrations, and **(i**) DTN versus DTP concentrations in the surface waters of Tongyeong (A region) during the summers of 2014, 2016, and 2017. The red filled circles denote the stations in the outbreak period of red tides. The red open-circled stations denote the center of the red-tide patches. The dashed lines indicate the Redfield ratio (N:P = 16).

In the surface waters of red-tide initiation region, A, salinities ranged from 29.98 to 34.13 throughout all study periods (Fig. 2). In general, the salinities in August (avg. 32.19 ± 0.97) and September (avg. 31.73 ± 0.35) were relatively lower than those in July (avg. 33.55 ± 0.37), for all study years. The lower salinities in this period are known to be associated with the CDW (salinity: <32)^15^. However, the lower-salinity waters in the red-tide regions are also influenced by stream water and groundwater, which are enriched in nutrients^11–13^.

In the surface waters of A region, the concentrations of chlorophyll *a* (chl. *a*) and peridinin (a diagnostic marker for dinoflagellates) during the red-tide period in 2014 were in the range of 1.3–7.2 µg L^−1^ and 0.4–5.7 µg L^−1^, respectively, which were much higher than those during the non-outbreak period in 2017 (Supplementary Fig. S1). In particular, the concentrations of chl. *a* and peridinin in the patch areas were 2- and 4-fold higher than those in the non-patch areas. In contrast, the concentrations of fucoxanthin (a diagnostic marker for diatoms) during the red-tide period (0.5–1.9 µg L^−1^) were lower than those during the non-outbreak period (Supplementary Fig. S1). As such, in the long-range spread region, B, the concentrations of chl. *a* and peridinin in the patch areas were 4- and 6-fold higher than those in the non-patch areas (Supplementary Fig. S1). This pigment trend is similar to that observed in previous red-tide periods in 2007^13^ and 2013^21^ in this region.

During the red-tide period in 2014, the concentrations of DIN and DIP in the surface waters of A region were in the range of 0.6–2.8 µM (avg. 1.5 ± 0.7 µM) and 0.06–0.26 µM (avg. 0.15 ± 0.07 µM), respectively (Fig. 2). These levels were much lower than those of DON (avg. 7.3 ± 1.6 μM) and DOP (avg. 0.33 ± 0.10 μM) for the same samples (Fig. 2). During the non-outbreak periods of red tides (2016 and 2017), the concentrations of DIN (avg. 2.0 ± 0.9 µM) and DIP (avg. 0.19 ± 0.04 µM) in the surface waters were similar to those in 2014, while the concentrations of DON (avg. 4.8 ± 0.8 µM) and DOP (avg. 0.24 ± 0.05 µM) were lower than those in 2014 (Fig. 2). For all study periods, the DIN/DIP ratios were lower and the DON/DOP ratios were higher than the Redfield ratio (16), and the DTN/DTP ratios were close to 16 (Fig. 2). In general, the DIN/DIP ratios in this region are lower than the Redfield ratio^13^. However, the DTN/DTP ratios seem to be similar to the Redfield ratio since the DON/DOP ratios were higher than the Redfield ratio as the turnover rate of nitrogen is more rapid^22,23^.

This trend suggests that the contributions of DON and DOP to the DTN and DTP were dominant (about 73 ± 10% for DTN and about 57 ± 9% for DTP) over all study periods. It is notable that the red-tide period had lower inorganic nutrients but higher organic and total nutrients than the non-outbreak periods. This trend is consistent with previous studies in other red-tide regions, including Oenarodo region of Korea^12,13^, Chesapeake Bay of USA^24^, and New York estuaries of USA^25^. In culture experiments, *C. polykrikoides* is capable of utilizing a variety of organic nitrogen compounds to maintain the growth under DIN limitation^24,25^. In addition, *C. polykrikoides* cultures grown on organic nitrogen compounds, such as glutamic acid and urea, showed significantly faster growth rates than cultures grown on NO_3_^−^ and NH_4_^+^ ^25^. However, diatoms are unable to utilize organic nutrients^26,27^. Thus, in general, red-tide outbreak is initiated by the rapid growth of dinoflagellates in competition with diatoms under the condition of depleted DIN or DIP and enriched organic nutrients as the supply of inorganic nutrients is halted^12,13,28,29^. In this study region, the outbreak of red tides occurs generally in outer areas of bays, which receive inner-bay waters with diatom blooms following high inorganic nutrient inputs^12–14^. This mechanism of red-tide outbreak has been well documented by previous studies in this region^12–14^.

**Figure 3:**
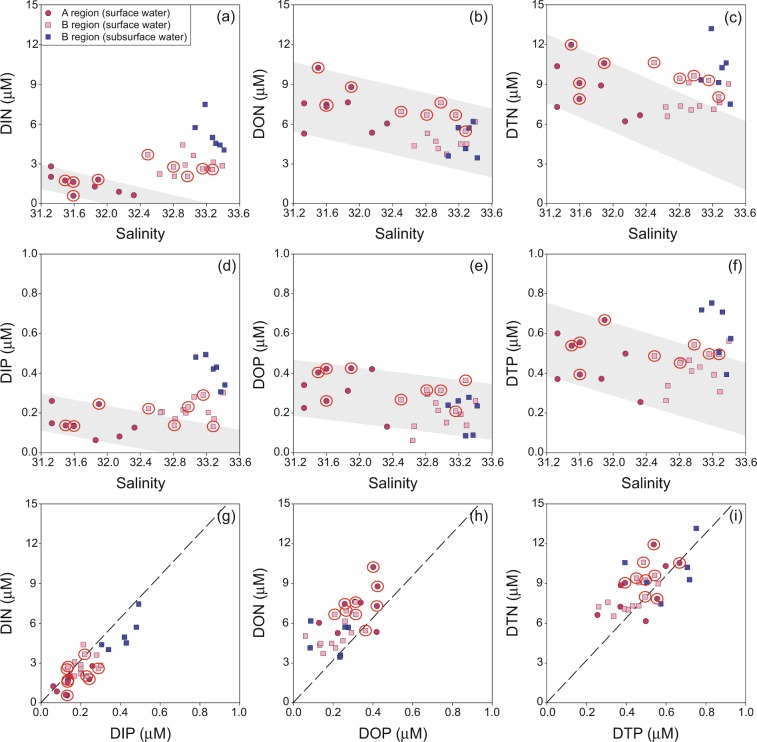
Scatter plots of salinities versus (**a**) DIN, (**b**) DON, (**c**) DTN, (**d**) DIP, (**e**) DOP, (**f**) DTP concentrations, (**g**) DIN versus DIP concentrations, (**h**) DON versus DOP concentrations, and (**i**) DTN versus DTP concentrations in the coasts of Tongyeong (A region) and Yeongdeok (B region) during the summer of 2014. The red open-circled stations are the center of the red-tide patches. The dashed lines indicate the Redfield ratio (N:P = 16). The shaded areas represent the ranges of nutrient concentrations expected from conservative mixing between A region and B region.

In the long-range spread region, B, the concentrations of DIN and DIP in the surface waters ranged from 2.0 to 4.4 µM (avg. 2.8 ± 0.7 µM) and from 0.13 to 0.30 µM (avg. 0.21 ± 0.05 µM), respectively, which were higher than those in the red-tide initiation region, A, but lower than those in the subsurface waters of B region (Fig. 3). This trend suggests that there were other inorganic-nutrient source inputs during the long-range water transport to B region. The concentrations of DON (avg. 5.4 ± 1.2 µM) and DOP (avg. 0.23 ± 0.08 µM) in the surface waters of B region were slightly lower than those in A region, but similar to those in the subsurface waters (Fig. 3). This trend suggests that the main source of organic nutrients are mainly from A region, together with local production (Fig. 3). The concentrations of DTN and DTP in the surface waters of B region were lower than those in A region or the subsurface waters of B region (Fig. 3).

### Tracing the nutrient sources using Ra isotopes

**Figure 4:**
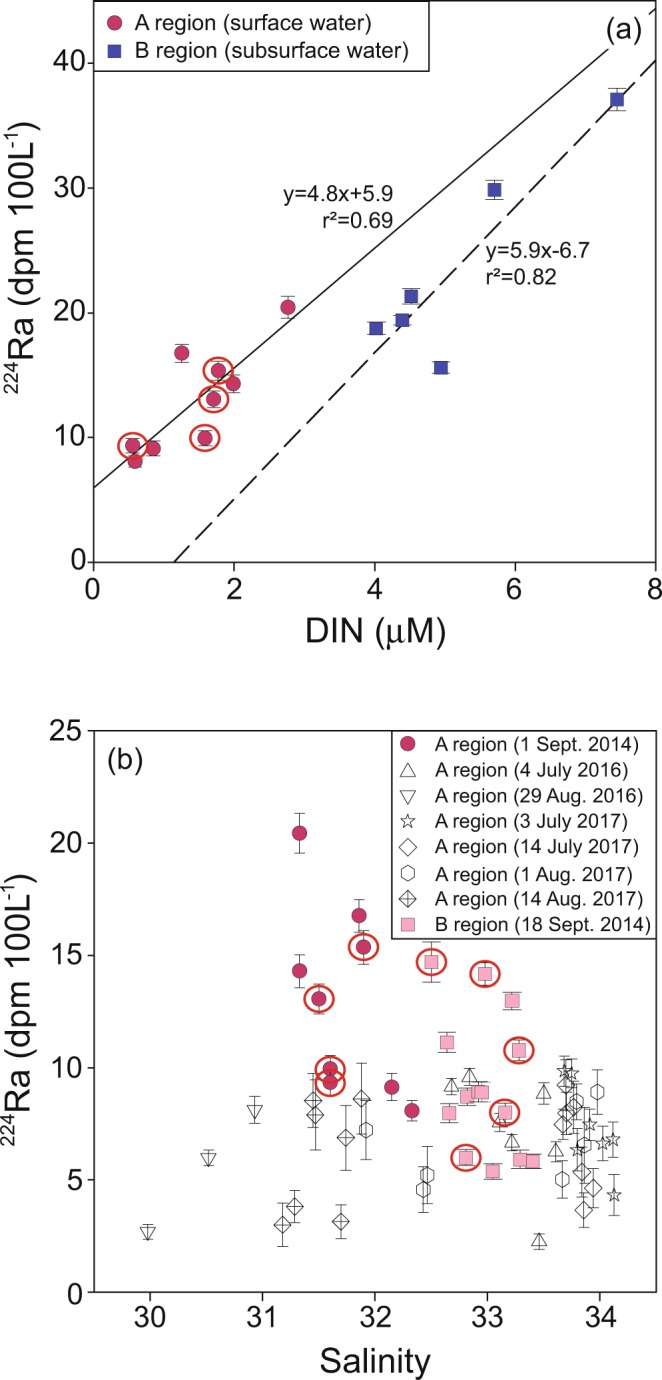
Scatter plots of ^224^Ra versus (**a**) DIN concentrations and (**b**) salinities in the coasts of Tongyeong (A region) and Yeongdeok (B region) during the summers of 2014, 2016, and 2017. The solid and dashed lines represent the relationships between DIN and ^224^Ra concentrations in the surface waters of A region and subsurface waters of B region, respectively. The red open-circled stations denote the center of the red-tide patches.

In general, the activities of ^223^Ra and ^224^Ra are enriched in coastal groundwater, river water, and bottom waters of the ocean due to their rapid production from their parents, ^228^Th and ^227^Ac, respectively, which are enriched in earth’s crust and sediments. Since Ra isotopes are very conservative and decay over time in seawater^30,31^, they have been utilized as a powerful tracer of nutrients which are similarly enriched in groundwater, river water, and deep waters of the ocean. In the study region, Ra isotopes have been used as an excellent tracer of groundwater-borne nutrient fluxes and nutrient sources fueling red tides^11–13^. As such, the activities of ^224^Ra correlated well with DIN concentrations in the surface waters of A region and with DIN concentrations in the subsurface waters of B region (Fig. 4a), indicating that Ra isotopes can be used as a tracer of nutrients in this region although nutrients are not conservative in offshore waters by biological utilization.

**Figure 5:**
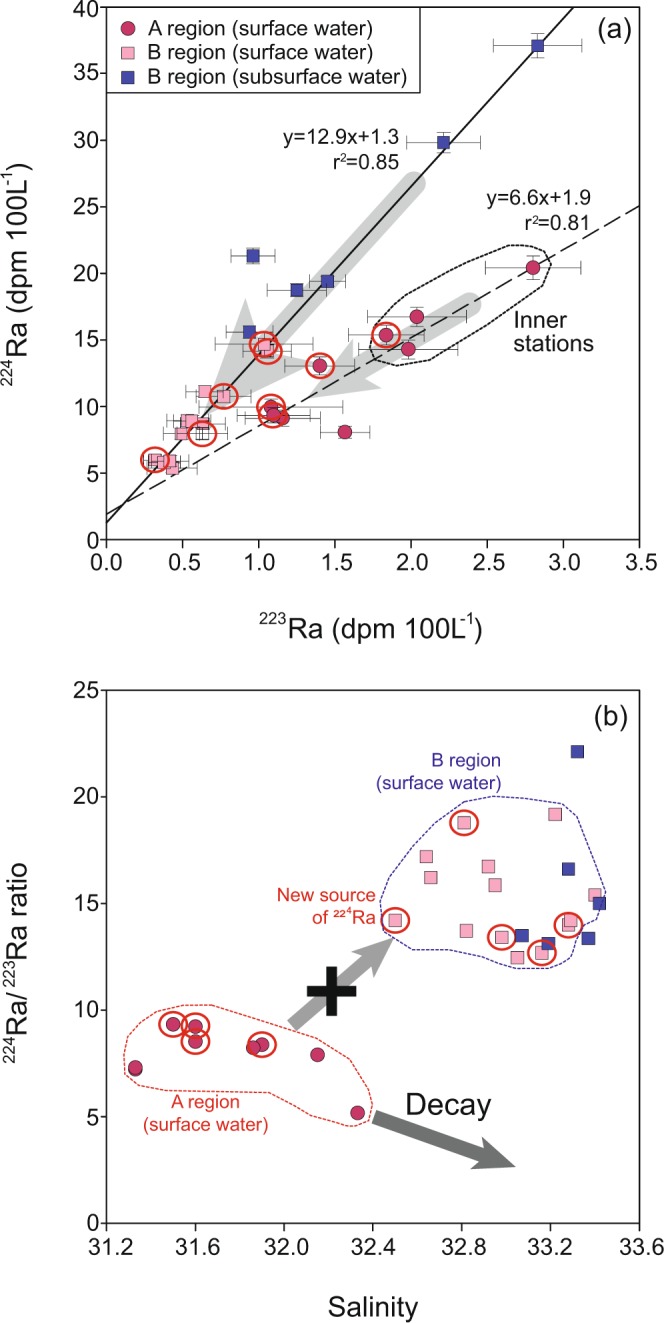
Scatter plots of (**a**) ^224^Ra versus ^223^Ra activities and (**b**) ^224^Ra/^223^Ra activity ratios versus salinities in the coasts of Tongyeong (A region) and Yeongdeok (B region) during the summer of 2014. The solid and dashed lines represent the relationships between ^224^Ra and ^223^Ra activities in the surface waters of A region and subsurface waters of B region, respectively. The gray filled arrow represents the supply of ^224^Ra in A region and B region. The red open-circled stations denote the center of the red-tide patches.

During the red-tide period in 2014, the activities of ^224^Ra in the surface waters of A region ranged from 8 to 20 dpm 100 L^−1^ (avg. 13 ± 4 dpm 100 L^−1^), which were much higher than those (2–10 dpm 100 L^−1^, avg. 7 ± 2 dpm 100 L^−1^) during the non-outbreak periods in 2016 and 2017 (Fig. 4b). This result is consistent with the previous results^12,13^ that the intensity of red tides in Oenarodo region of Korea is controlled by the magnitude of groundwater-borne nutrient inputs in the red-tide region as traced by ^224^Ra. Although inorganic nutrients are depleted in the red-tide region (Fig. 2), higher ^224^Ra activities in the red-tide region confirm that the larger supply of inorganic nutrients in the massive red-tide outbreak in Tongyeong (A region) is also critical (Fig. 4b). In B region, the activities of ^224^Ra in the surface waters ranged from 5 to 15 dpm 100 L^−1^ (avg. 9 ± 3 dpm 100 L^−1^) (Fig. 4b), which were lower than those in the surface waters of A region and the subsurface waters of B region (Fig. 5a).

In order to differentiate nutrient sources, we plotted ^224^Ra versus ^223^Ra activities in the red-tide initiation region (A) versus the spread region (B) (Fig. 5a). This plot clearly shows that the main source of Ra (together with nutrients) in A region is from inshore waters, which may be associated with coastal nutrient inputs through surface runoffs and/or submarine groundwater discharge, as shown in Oenarodo region of Korea^11–13^. In contrast, the main source of nutrients in B region seems to be from the subsurface waters. The ^224^Ra/^223^Ra ratios in the surface waters of B region were higher than those in the surface waters of A region but similar to those in the subsurface waters of B region (Fig. 5b). If the main source of ^224^Ra is from A region, ^224^Ra/^223^Ra ratios should be a few times lower than those in the source region due to rapid decay of ^224^Ra (half-life: 3.4 days) relative ^223^Ra (half-life: 11.3 days) during the travel period of about 20 days (Fig. 5b). This conclusion is also supported by ^224^Ra/^223^Ra ratios against salinities as the slope between ^223^Ra and ^224^Ra activities in B region is different from that in A region (Fig. 5a).

The southerly winds prevailed over the eastern coast of Korea during early- and mid-September, 2014 (https://data.kma.go.kr). This wind condition might cause coastal upwelling along the eastern coast of Korea as evidenced by a sharp decrease in surface temperature (about 2 °C) between 8 and 19 September at Pohang site, approximately 50 km south of B region (Supplementary Fig. S2). Although the occurrence of upwelling is uncertain, the upwelling or vertical mixing between the surface and subsurface layers seemed to be rapid enough to pump nutrients to the surface layer without the significant decay of ^224^Ra. Thus, our results suggest that the spread of red tides has been fueled by dissolved inorganic nutrients from the subsurface waters, perhaps due to coastal upwelling, in addition to the organic nutrients.

## Conclusions

Short-lived naturally occurring Ra isotopes (^223^Ra and ^224^Ra) can successfully trace the sources of nutrients fueling initiation and spread of red tides along the coast of Korea during the summer of 2014. The offshore red-tide areas in the study region showed low-inorganic and high-organic nutrient concentrations. Based on Ra isotopes, we reveal that the main source of nutrients fueling red-tide initiation is transported horizontally from inner-shore waters. However, the spread of red tides seems to be supported by dissolved inorganic nutrients from the subsurface waters, in addition to the organic nutrients. We conclude that the supply of nutrients from various pathways is critical to maintain massive red tides, and the outbreak of red tides is associated with compositions of inorganic and organic nutrients. Thus, our research tools can be used in other red-tide areas of the world in order to determine major nutrient sources and spread mechanisms of red tides.

## Supplementary information

Supplementary information

## Data Availability

The datasets analyzed during the current study are available from the corresponding author on reasonable request.
