# Supplementary material for: Tracing the sources of nutrients fueling dinoflagellate red tides occurring along the coast of Korea using radium isotopes
Source: Sci Rep. 2019 Oct 25;9:15319. doi: 10.1038/s41598-019-51623-w (PMC6814798; doi:10.1038/s41598-019-51623-w)
Supplement: Supplementary file 1 — Supplementary information [file 41598_2019_51623_MOESM1_ESM.docx]

**Supplementary Information**

**Tracing the sources of nutrients fueling dinoflagellate red tides occurring along the coast of Korea using radium isotopes**

Hyeong Kyu Kwon^1^, Guebuem Kim^1*^, Yongjin Han^1^, Junhyeong Seo^1^, Weol Ae Lim^2^, Jong Woo Park^2^, Tae Gyu Park^3^ & In-Seong Han^2^

^1^School of Earth and Environmental Sciences/Research Institute of Oceanography, Seoul National University, Seoul 08826, Republic of Korea

^2^Ocean Climate and Ecology Research Division, National Institute of Fisheries Science, Busan 46083, Republic of Korea

^3^Southeast Sea Fisheries Research Institute, National Institute of Fisheries Science, Tongyeong 53085, Republic of Korea

^*^Corresponding author: Guebuem Kim (gkim@snu.ac.kr)


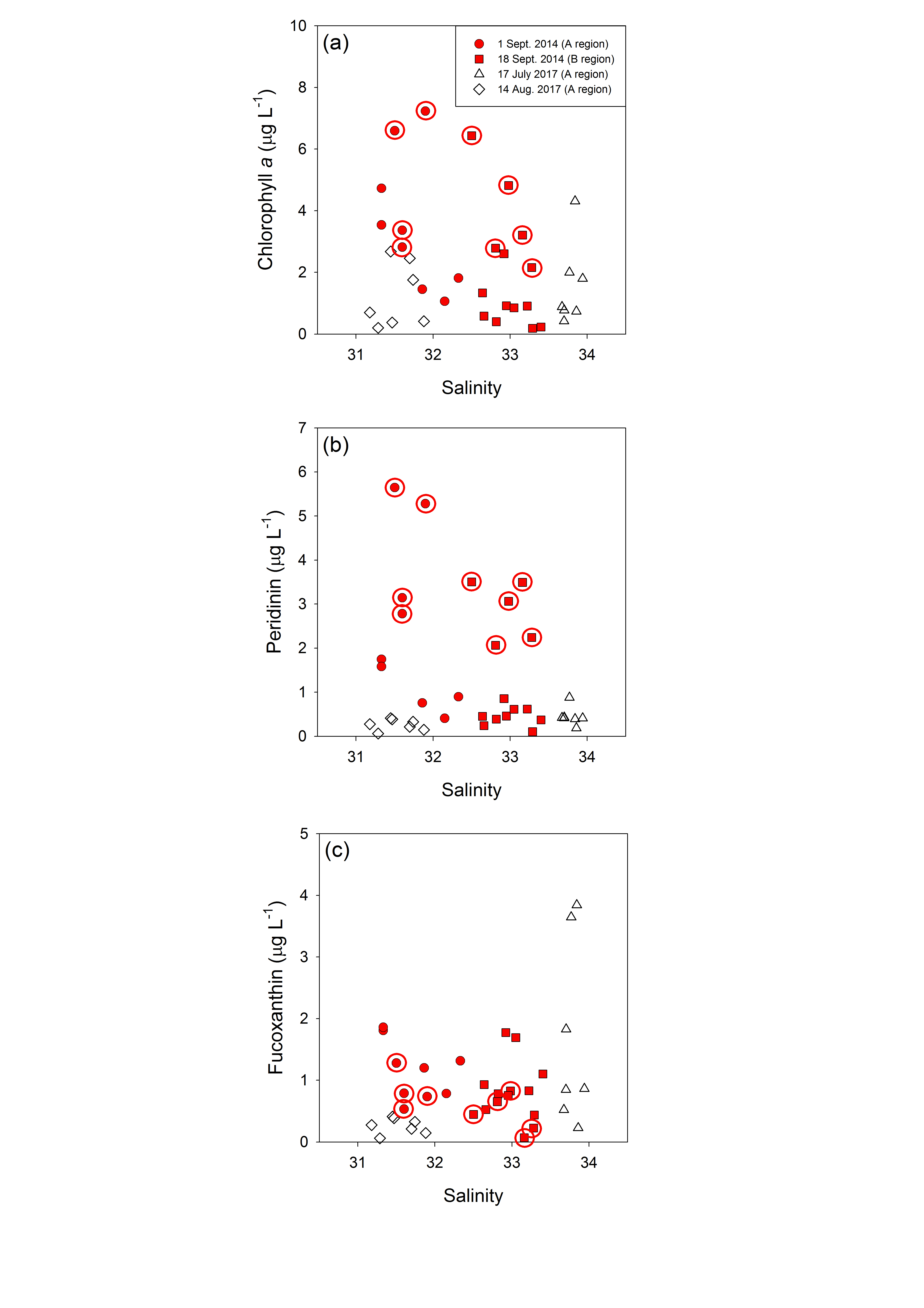


**Figure S1.** Scatter plots of salinities versus (a) chlorophyll *a*, (b) peridinin, and (c) fucoxanthin concentrations in the surface waters of Tongyeong (A region) and Yeongkeok (B region) during the summers of 2014 and 2017. The red open-circled stations denote the center of the red-tide patches.


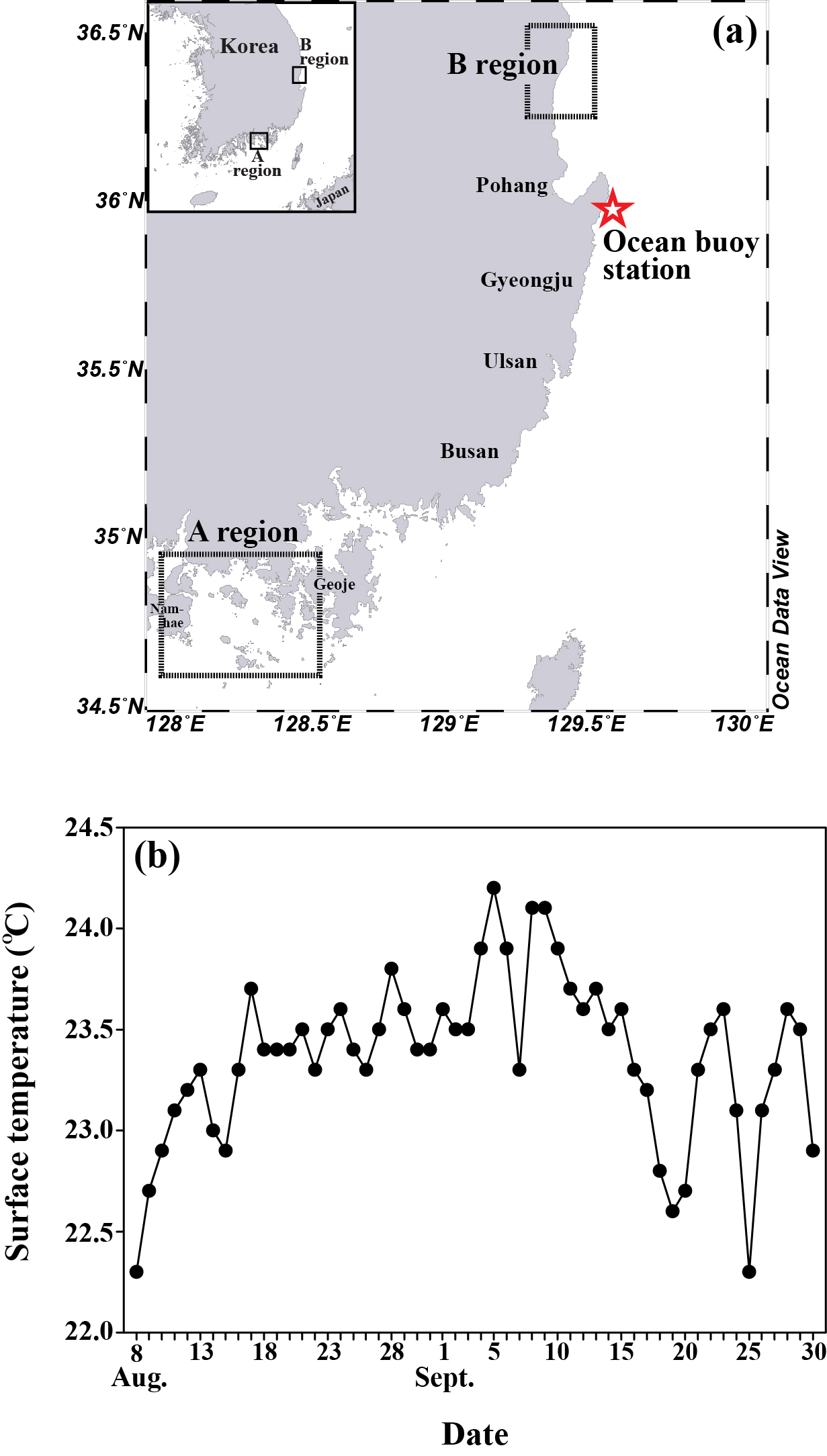


**Figure S2.** (a) A location map of the ocean buoy station in Pohang site in the eastern coast of Korea. (b) The daily variation of surface temperature at the buoy station in Pohang site from August 8 to September 30, 2014.

**Table S1.** The concentrations of salinity, chlorophyll *a*, peridinin, fucoxanthhin, DIN, DON, DIP, DOP, ^223^Ra, and ^224^Ra in the surface waters off Tongyeong (A region) in the southern coast of Korea during the summers of 2014, 2016, and 2017.

| **Station** | | **Salinity** | **Pigments (μg L^-1^)** | | | **Nutrients (μM)** | | | | **Ra isotopes (dpm 100L^-1^)** | |
| --- | --- | --- | --- | --- | --- | --- | --- | --- | --- | --- | --- |
|  |  |  | **Chl. *a*** | **Peri** | **Fuco** | **DIN** | **DON** | **DIP** | **DOP** | **^223^Ra** | **^224^Ra** |
| Sept.  1-2,  2014 | T1 | 31.33 | 3.5 | 1.7 | 1.8 | 2.0 | 5.3 | 0.15 | 0.22 | 2.0±0.3 | 14.3±0.7 |
|  | T2 | 31.50 | 6.6 | 5.6 | 1.3 | 1.7 | 10.2 | 0.14 | 0.40 | 1.4±0.2 | 13.1±0.7 |
|  | T3 | 31.60 | 3.4 | 3.1 | 0.8 | 1.6 | 7.5 | 0.13 | 0.26 | 1.1±0.5 | 9.9±0.6 |
|  | T4 | 32.15 | 1.1 | 0.4 | 0.8 | 0.8 | 5.3 | 0.08 | 0.42 | 1.2±0.2 | 9.1±0.6 |
|  | T5 | 31.33 | 4.7 | 1.6 | 1.9 | 2.8 | 7.5 | 0.26 | 0.34 | 2.8±0.3 | 20.4±0.9 |
|  | T6 | 31.90 | 7.2 | 5.3 | 0.7 | 1.8 | 8.8 | 0.24 | 0.42 | 1.8±0.2 | 15.4±0.8 |
|  | T7 | 31.60 | 2.8 | 2.8 | 0.5 | 0.6 | 7.3 | 0.13 | 0.42 | 1.1±0.2 | 9.3±0.6 |
|  | T8 | 32.33 | 1.8 | 0.9 | 1.3 | 0.6 | 6.0 | 0.13 | 0.13 | 1.6±0.2 | 8.1±0.5 |
|  | T9 | 31.86 | 1.4 | 0.8 | 1.2 | 1.3 | 7.6 | 0.06 | 0.31 | 2.0±0.3 | 16.7±0.7 |
| July  4-5,  2016 | T2 | 33.50 | - | - | - | 2.8 | 4.7 | 0.18 | 0.20 | 2.7±0.2 | 8.8±0.5 |
|  | T4 | 33.61 | - | - | - | 1.2 | 4.2 | 0.19 | 0.24 | 1.3±0.1 | 6.3±0.4 |
|  | T5 | 32.84 | - | - | - | 2.8 | 4.2 | 0.29 | 0.21 | 1.7±0.2 | 9.6±0.4 |
|  | T8 | 33.46 | - | - | - | 1.4 | 4.2 | 0.13 | 0.14 | 0.5±0.0 | 2.3±0.4 |
|  | T9 | 32.68 | - | - | - | 2.3 | 4.4 | 0.23 | 0.28 | 1.1±0.2 | 9.2±0.4 |
|  | T10 | 33.11 | - | - | - | 2.7 | 4.9 | 0.16 | 0.28 | 1.5±0.2 | 7.6±0.4 |
|  | T11 | 33.22 | - | - | - | 1.2 | 3.2 | 0.13 | 0.28 | 1.0±0.1 | 6.7±0.4 |
| July  18-19,  2016 | T2 | 33.25 | - | - | - | 2.2 | 3.5 | 0.13 | 0.31 | - | - |
|  | T4 | 33.43 | - | - | - | 2.8 | 5.2 | 0.17 | 0.30 | - | - |
|  | T5 | 33.13 | - | - | - | 1.5 | 4.5 | 0.16 | 0.29 | - | - |
|  | T8 | 33.23 | - | - | - | 1.0 | 5.1 | 0.16 | 0.30 | - | - |
|  | T9 | 33.18 | - | - | - | 1.1 | 4.4 | 0.17 | 0.26 | - | - |
|  | T10 | 33.46 | - | - | - | 1.4 | 5.7 | 0.18 | 0.25 | - | - |
|  | T11 | 33.37 | - | - | - | 1.7 | 4.9 | 0.18 | 0.21 | - | - |
| Aug.  1-2,  2016 | T2 | 33.09 | - | - | - | 2.8 | 6.2 | 0.20 | 0.26 | - | - |
|  | T4 | 33.48 | - | - | - | 1.3 | 4.8 | 0.21 | 0.23 | - | - |
|  | T5 | 32.98 | - | - | - | 1.2 | 5.1 | 0.23 | 0.23 | - | - |
|  | T8 | 32.28 | - | - | - | 0.7 | 4.2 | 0.22 | 0.28 | - | - |
|  | T9 | 32.79 | - | - | - | 0.9 | 5.2 | 0.26 | 0.25 | - | - |
|  | T10 | 32.12 | - | - | - | 1.4 | 5.0 | 0.23 | 0.29 | - | - |
|  | T11 | 31.60 | - | - | - | 0.9 | 6.7 | 0.23 | 0.29 | - | - |
| Aug.  16-17,  2016 | T2 | 32.35 | - | - | - | 3.2 | 3.1 | 0.15 | 0.27 | - | - |
|  | T4 | 31.79 | - | - | - | 0.9 | 3.8 | 0.19 | 0.26 | - | - |
|  | T5 | 32.48 | - | - | - | 0.8 | 5.0 | 0.19 | 0.26 | - | - |
|  | T8 | 32.25 | - | - | - | 2.1 | 4.0 | 0.18 | 0.27 | - | - |
|  | T9 | 32.86 | - | - | - | 2.0 | 5.6 | 0.21 | 0.26 | - | - |
|  | T10 | 31.83 | - | - | - | 1.9 | 4.4 | 0.17 | 0.24 | - | - |
|  | T11 | 31.85 | - | - | - | 1.2 | 5.2 | 0.18 | 0.26 | - | - |
| Aug. 29,  2016 | T5 | 30.93 | - | - | - | 2.5 | 4.1 | 0.18 | 0.14 | 1.1±0.3 | 8.1±0.6 |
|  | T9 | 30.52 | - | - | - | 2.5 | 4.9 | 0.18 | 0.18 | 1.2±0.2 | 6.0±0.3 |
|  | T10 | 29.98 | - | - | - | 1.9 | 5.0 | 0.19 | 0.17 | 0.4±0.1 | 2.7±0.3 |
| July  3-4,  2017 | T2 | 33.91 | - | - | - | 2.4 | 5.6 | 0.20 | 0.23 | 0.7±0.2 | 7.5±0.7 |
|  | T4 | 34.12 | - | - | - | 2.3 | 3.7 | 0.21 | 0.21 | 1.1±0.4 | 6.8±0.8 |
|  | T5 | 33.68 | - | - | - | 3.8 | 5.3 | 0.20 | 0.27 | 1.2±0.2 | 9.8±0.7 |
|  | T8 | 34.03 | - | - | - | 2.4 | 5.3 | 0.21 | 0.25 | 0.8±0.4 | 6.6±0.8 |
|  | T9 | 33.75 | - | - | - | 3.2 | 5.9 | 0.23 | 0.26 | 1.8±0.4 | 9.7±0.6 |
|  | T10 | 33.80 | - | - | - | 2.4 | 5.2 | 0.17 | 0.32 | 1.1±0.2 | 6.3±0.9 |
|  | T11 | 34.13 | - | - | - | 2.4 | 3.8 | 0.18 | 0.24 | 1.2±0.2 | 4.3±0.9 |

**Table S1.** Continued.

| **Station** | | **Salinity** | **Pigments (μg L^-1^)** | | | **Nutrients (μM)** | | | | **Ra isotopes (dpm 100L^-1^)** | |
| --- | --- | --- | --- | --- | --- | --- | --- | --- | --- | --- | --- |
|  |  |  | **Chl. *a*** | **Peri** | **Fuco** | **DIN** | **DON** | **DIP** | **DOP** | **^223^Ra** | **^224^Ra** |
| July  17-18,  2017 | T2 | 33.70 | 0.4 | 0.4 | 0.8 | 2.2 | 4.8 | 0.21 | 0.27 | 1.7±0.3 | 9.2±1.1 |
|  | T4 | 33.94 | 1.8 | 0.4 | 0.9 | 1.7 | 5.3 | 0.23 | 0.16 | 0.9±0.2 | 4.6±0.9 |
|  | T5 | 33.67 | 0.9 | 0.4 | 0.5 | 2.9 | 4.2 | 0.15 | 0.13 | 1.0±0.2 | 7.5±0.7 |
|  | T8 | 33.77 | 2.0 | 0.9 | 3.6 | 1.6 | 5.2 | 0.16 | 0.19 | 0.9±0.2 | 8.2±1.2 |
|  | T9 | 33.70 | 0.4 | 0.4 | 1.8 | 2.5 | 4.9 | 0.19 | 0.28 | 1.2±0.3 | 8.0±0.9 |
|  | T10 | 33.84 | 4.3 | 0.4 | 3.8 | 1.9 | 5.5 | 0.18 | 0.15 | 1.3±0.2 | 5.3±1.1 |
|  | T11 | 33.86 | 0.7 | 0.2 | 0.2 | 1.3 | 5.3 | 0.28 | 0.19 | 1.3±0.2 | 3.7±0.8 |
| Aug.  1-2,  2017 | T2 | 33.86 | - | - | - | 3.5 | 4.8 | 0.20 | 0.26 | 2.1±0.3 | 6.5±1.7 |
|  | T4 | 33.67 | - | - | - | 2.5 | 4.2 | 0.21 | 0.23 | 1.2±0.2 | 5.0±0.8 |
|  | T5 | 33.98 | - | - | - | 4.8 | 4.1 | 0.30 | 0.23 | 1.5±0.3 | 8.9±1.0 |
|  | T8 | 32.43 | - | - | - | 1.7 | 4.7 | 0.17 | 0.28 | 0.3±0.1 | 4.6±1.0 |
|  | T9 | 33.79 | - | - | - | 4.0 | 5.0 | 0.30 | 0.25 | 1.4±0.3 | 8.5±0.8 |
|  | T10 | 32.46 | - | - | - | 1.3 | 2.8 | 0.16 | 0.21 | 0.7±0.2 | 5.2±1.3 |
|  | T11 | 31.92 | - | - | - | 2.3 | 4.8 | 0.17 | 0.29 | 0.2±0.1 | 7.2±1.3 |
| Aug.  14-15,  2017 | T2 | 31.74 | 1.8 | 0.3 | 2.4 | 1.7 | 4.3 | 0.24 | 0.24 | 0.7±0.2 | 6.9±1.4 |
|  | T4 | 31.45 | 2.7 | 0.4 | 3.4 | 1.7 | 6.1 | 0.23 | 0.24 | 0.1±0.1 | 8.5±1.2 |
|  | T5 | 31.88 | 0.4 | 0.1 | 0.7 | 3.1 | 4.2 | 0.25 | 0.23 | 1.0±0.2 | 8.6±1.6 |
|  | T8 | 31.70 | 2.5 | 0.2 | 2.4 | 1.6 | 5.7 | 0.21 | 0.25 | 0.6±0.2 | 3.1±0.8 |
|  | T9 | 31.29 | 0.2 | 0.1 | 1.0 | 2.6 | 5.2 | 0.15 | 0.30 | 0.3±0.2 | 3.8±0.7 |
|  | T10 | 31.47 | 0.4 | 0.4 | 0.9 | 1.0 | 5.0 | 0.12 | 0.22 | 0.8±0.2 | 7.9±1.6 |
|  | T11 | 31.18 | 0.7 | 0.3 | 0.6 | 0.6 | 5.3 | 0.14 | 0.19 | 0.2±0.1 | 3.0±1.0 |

**Table S2.** The concentrations of salinity, , chlorophyll *a*, peridinin, fucoxanthhin, DIN, DON, DIP, DOP, ^223^Ra, and ^224^Ra in the surface and subsurface waters off Yeongdeok (B region) in the eastern coast of Korea in September 18-19, 2014.

| **Station** | | **Salinity** | **Pigments (μg L^-1^)** | | | **Nutrients (μM)** | | | | **Ra isotopes (dpm 100L^-1^)** | |
| --- | --- | --- | --- | --- | --- | --- | --- | --- | --- | --- | --- |
|  |  |  | **Chl. *a*** | **Peri** | **Fuco** | **DIN** | **DON** | **DIP** | **DOP** | **^223^Ra** | **^224^Ra** |
| Surface  water | Y1 | 32.50 | 6.4 | 3.5 | 0.4 | 3.7 | 6.9 | 0.22 | 0.27 | 1.0±0.3 | 14.7±0.9 |
|  | Y2 | 32.98 | 4.8 | 3.1 | 0.8 | 2.0 | 7.6 | 0.23 | 0.31 | 1.1±0.2 | 14.2±0.5 |
|  | Y3 | 33.05 | 0.9 | 0.6 | 1.7 | 3.6 | 3.7 | 0.28 | 0.15 | 0.4±0.2 | 5.4±0.3 |
|  | Y4 | 32.92 | 2.6 | 0.9 | 1.8 | 4.4 | 4.7 | 0.21 | 0.25 | 0.5±0.1 | 8.9±0.5 |
|  | Y5 | 32.95 | 0.9 | 0.5 | 0.8 | 2.9 | 4.1 | 0.20 | 0.21 | 0.6±0.1 | 8.9±0.4 |
|  | Y6 | 33.16 | 3.2 | 3.5 | 0.1 | 2.6 | 6.7 | 0.29 | 0.21 | 0.6±0.2 | 8.0±0.4 |
|  | Y7 | 32.64 | 1.3 | 0.5 | 0.9 | 2.2 | 5.0 | 0.20 | 0.06 | 0.6±0.1 | 11.1±0.5 |
|  | Y8 | 32.81 | 2.8 | 2.1 | 0.7 | 2.7 | 6.7 | 0.14 | 0.31 | 0.3±0.1 | 6.0±0.3 |
|  | Y9 | 32.66 | 0.6 | 0.2 | 0.5 | 2.2 | 4.4 | 0.20 | 0.13 | 0.5±0.1 | 8.0±0.4 |
|  | Y10 | 33.22 | 0.9 | 0.6 | 0.8 | 2.6 | 4.5 | 0.20 | 0.19 | 0.7±0.1 | 13.0±0.4 |
|  | Y11 | 32.82 | 0.4 | 0.4 | 0.8 | 2.0 | 5.3 | 0.17 | 0.29 | 0.6±0.1 | 8.7±0.4 |
|  | Y12 | 33.40 | 0.2 | 0.4 | 1.1 | 2.8 | 6.2 | 0.30 | 0.26 | 0.4±0.1 | 5.8±0.3 |
|  | Y13 | 33.28 | 2.2 | 2.2 | 0.2 | 2.5 | 5.4 | 0.13 | 0.36 | 0.8±0.2 | 10.8±0.4 |
|  | Y14 | 33.29 | 0.2 | 0.1 | 0.4 | 3.1 | 4.5 | 0.17 | 0.14 | 0.4±0.1 | 5.9±0.4 |
| Subsurface  water | Y1 (10 m) | 33.07 | - | - | - | 5.7 | 3.6 | 0.48 | 0.24 | 2.2±0.2 | 29.8±0.8 |
|  | Y2 (10 m) | 33.19 | - | - | - | 7.5 | 5.7 | 0.49 | 0.26 | 2.8±0.3 | 37.1±0.9 |
|  | Y6 (20 m) | 33.32 | - | - | - | 4.5 | 5.7 | 0.43 | 0.28 | 1.0±0.1 | 21.3±0.6 |
|  | Y7 (10 m) | 33.28 | - | - | - | 4.9 | 4.1 | 0.42 | 0.08 | 0.9±0.2 | 15.6±0.4 |
|  | Y10 (20 m) | 33.42 | - | - | - | 4.0 | 3.4 | 0.34 | 0.23 | 1.3±0.2 | 18.7±0.5 |
|  | Y13 (10 m) | 33.37 | - | - | - | 4.4 | 6.2 | 0.31 | 0.09 | 1.5±0.1 | 19.4±0.4 |
